# Past—Present—Future: Urban Spatial Succession and Transition of Rail Transit Station Zones in Japan

**DOI:** 10.3390/ijerph192013633

**Published:** 2022-10-20

**Authors:** Xinyu Zhuang, Li Zhang, Jie Lu

**Affiliations:** 1 College of Quality & Standardization, Qingdao University, Qingdao 266071, China; 2 Department of Architecture and Urban Design, Faculty of Human-Environment Studies, Kyushu University, Fukuoka 819-0395, Japan; 3 BMP Construction Consulting (Shanghai) Co., Ltd., Shanghai 200336, China; 4 Qingdao Institute of Standardization, Qingdao 266101, China

**Keywords:** Japan rail transit, land use, urban spatial succession, urban regeneration, sustainable development

## Abstract

In today’s environmentally conscious society, advocated by a global point of view, land and building use around rail transit stations have changed in the urbanization process. Promoting urban construction and development centered on rail transit stations not only meets the actual needs of urban sustainable development but is also an important means to guide the development of innovative cities. Therefore, it is meaningful to study the characteristics of urban spatial succession, development rules, and future trends based on this new perspective. We analyzed the relationship between rail transit networks and urban form in Japan using GIS by investigating changes in land and building use around rail transit stations over 30 years in the 1980s, 1990s and 2000s (from 1985–2010) using factor analysis and cluster analysis, and we discussed the impact of land consolidation planning and the setting and site selection of new stations based on urban development to understand the development trends inside and outside station zones and urban spatial succession. The results showed the following: (1) There are certain relationships between the development of urban form and traffic demand, and the rail transit network in Japan has the characteristics of high accessibility and aggregation of a small network; (2) Commercial development with a high plot ratio is dispersed and diverted by high-density rail transit stations in the commercial center of Japan; and (3) Commercial sub-centers form complexes by integrating multi-line transfers and form regional linkages through clustered commercial development. Regional business centers realize the agglomeration and radiation of functions through the compound development of station zones. This case study on rail transit zones and urban spatial succession in Japan has important enlightenment significance for urban construction toward optimizing the location and development of suburban rail transit lines, promoting the compact development of cities, exploring new ways to build more reasonable transport, planning city design and layout for rail transit station zones, and providing decision-making references for urban regeneration and sustainable development.

## 1. Introduction

Cities grow with the development of human society, and modern urban spaces are evolving towards the future. Every city in the world has formed its own urban spatial characteristics since their beginning, and different urban spaces have different characteristics [1]. The formation, development, and evolution of urban spaces have their own rules. The evolution mode, scale, and process of cities in the world differ [2]. In the construction of modern urban spaces, it is particularly important to explore their evolution and development. How will urban spaces develop and evolve? How do we create future urban spaces? What are the non-negligible ways to understand, evaluate, and quantify the interactions among people’s activities, transportation, and the urban environment? These are questions worth paying attention to in urban sustainable development.

With the acceleration of global urbanization, traffic congestion and the urban environment have become bottlenecks that challenge the sustainability of cities. If the urban traffic problem cannot be effectively solved, it will seriously affect the development of cities [3,4,5]. In the 21st century, the construction of urban rail transits with green, low-carbon [6], energy-saving [7], and fast and large traffic volume [8] characteristics has attracted more attention. Modern society will continue to develop around public transportation, and the formation of urban texture based on rail transit is very important in the process of urban development.

Urban development has spread in a disorderly manner to surrounding areas in recent years. In order to promote the functional integration of cities, realizing closer connections with public transport networks, adjusting the urban structure, and compressing the urban scale have become top priorities [9]. In addition, the potential value of rail transit stations and the development of integrated urban stations is understood. With the development of land use around railways and subways, the problem of disordered urban development has become serious, not only in the central areas around stations, but also in sparsely populated areas. The changes in land use around rail transit stations represents the development of commerce, industry, agriculture, housing, parks and green spaces, urban facilities, etc. [10]. Due to the continuous development of land, these influencing elements will gradually lead to one or more radiation circles forming in the process of change, which will have diverse impacts on the development of cities and people’s lives. In this study, such areas are called “station zones”. In order to promote development inside and outside the station zones and adjust the disordered urban development, it is urgent to constantly explore new ways of developing rail transit.

The rail transit network in Japan is known throughout the world for its superiority and punctuality. Recently, with the development of urbanization, the value of rail transit stations is becoming more important. Railway stations are necessary links for a city, and city planning and policies for land use have been enforced by centering on rail transit stations. It is thought that urban development centered on rail transit stations is closer to urban planning centered on public traffic in an environment-oriented society [11]. In Japan, after the bubble economy in 1996, the development of areas surrounding station zones was somewhat slow, and the number of shops and restaurants within 0–400 m of station zones declined. Large-scale commercial facilities tended to relocate to the suburbs, and the original central business districts gradually went into a depression. Shopping streets around rail transit stations, which are composed of large retail stores and small shops, have become a serious problem affecting city development. This is a problem that has seriously affected not only small cities, but also central cities such as Fukuoka. In addition, people have paid more attention to urban environmental problems, and the improvement of environmental problems around railways has gradually increased. 

Under the guidance of local governments in Japan, renovation projects around rail transit stations have been gradually carried out. The Japanese government has increased land adjustment projects in Fukuoka, and it has developed rail transit stations and surrounding areas by actively building elevated roads and buildings. With the development of rail transit stations in Fukuoka, the relationship between stations and the surrounding environment is becoming closer [12]. Urban planning centered on public transport hubs has become increasingly prominent. Therefore, this study took rail transit stations and station zones in Japan as its research object, and it focused on the distribution of rail transit stations, the changes of station zones and their relationship with the population and passengers, station classification, and the spatial succession of rail transit stations and station zones over the years. We also analyzed the change characteristics of station zones in Japan over 30 years (from 1985–2010) in order to provide a reference for traffic planning, station selection, and urban spatial layout, to and promote the compact and orderly development of cities. The purposes of this study were as follows: (1) to explore a new research perspective focusing on typology analysis of urban clusters and to enrich correlation theory of urban space, urban growth and succession; (2) to propose a methodology for analyzing urban transition around rail transit stations and annual changes in station typification; (3) to find a way to study the relationships among people’s activities, transportation, and the urban environment, the data of the changes of land use around station zones can be used to reveal the response of transportation to environment changes, which can expose the theme further; and (4) to provide a well-founded reference on what happened as an outcome of transportation infrastructure improvement and urban transportation. 

Scholars have carried out much research in this area, but there are still areas to be explored. First, this is a longitudinal study, focusing on the period from 1985 to 2010 to show how transportation and land use has interacted over time, which includes three succession stages that represent the golden period of urban development, transition, and consolidation in Japan. This period has also had a far-reaching impact on urban succession. Second, few studies have examined station typification and changes on the transition modes and characteristics of rail transit zones, while this study explores the method of analysis of urban clusters, creating a typology based on land use mixes. This paper is organized as follows: Section 1 and Section 2 present a general introduction including the background, previous reviews, research purposes, and differences from previous studies. In Section 3 and Section 4, the research design and methodology are presented. The description of target lines and stations, the concept and division of station zones, which were the focus in this study, are defined. In Section 5, we analyze the changes of land use and development trends in the inner and outer areas of station zones spanning 30 years. Furthermore, we use factor analysis and cluster analysis of stations to find out the urban spatial succession and transition of rail transit station zones, which are clustered into groups and compared according to different types and characteristics among stations. Section 6 provides a discussion with theoretical contributions and practical implications, and the conclusions of this study are summarized in Section 7.

## 2. Literature Review

### 2.1. Study on the Development of Rail Transit

Urban rail transit (URT) is a public transport system that uses rail guidance and can include a subway system, light rail system, trams, monorail system, automatic guided rail system, rapid rail system, and maglev system [13]. Since smooth, efficient, and reliable transportation is the basis for passengers to choose a travel mode, urban rail transit has become an important part of public transport to alleviate traffic congestion because it is fast, convenient, and safe, can handle large volumes, and is highly efficient [14].

Over the years, some researchers have investigated the development of railways and subway stations; the relationship between urban development and stations, i.e., the theory of rail transit and development [15]; the impact on the land market and house prices [16]; changes in land and house prices [17]; and the influence of rail transit stations in urban development [18]. Sustainable development and a livable environment represent the big visionary ideas of urban planning, but there are also a host of conflicts to solve. Woudsma et al. [19] proposed that there is a connection between transportation and land use. Kim and Byun analyzed the impact of subway stations on commercial land value and used the methods of linear regression and regression analysis to determine the significant impact of subway stations on commercial land prices [20], and on this basis, established a model of the spatial relation between land price and distance. Bertolini et al. noted that the redevelopment of railway stations and surrounding areas has been high on the agenda of European cities for more than two decades [21]. Driving forces include the expansion and upgrading of rail infrastructure, the reduced demand for industrial space in central urban areas, the privatization of railways, efforts to increase the attractiveness of cities, the exploration of sustainable development, and the spatial dynamics of contemporary society. Ishida et al. researched areas of JR Osaka Station and collected data to devise a method for initiating evacuation, and they explained that developing rail transit and doing construction above subway stations would have a profound influence on the city [22]. Lee explored differences in the effects of rail transit investment across various types of land with different values and in different locations, using the development of Seoul Metro Line 9 (SML9) in Korea as a case study [23]. 

### 2.2. Study of Rail Transit Stations and Urban Space

The development and evolution of cities include the creation, preservation, protection, revitalization, negation, and development of urban space, and the reasoning and assumption in planning and design [24]. Urban space is mainly characterized by land use, buildings, and natural objects, as defined by urban development [25]. Thus, urban space can be defined as a purposeful external environment created by people’s activities. The growth of urban space is reflected not only in the expansion of land use, but also in spatial succession. The succession of land use can more profoundly reveal the characteristics of urban spatial expansion, which is a spatial reflection of urban functional agglomeration and diffusion. Finding ways to promote more reasonable succession modes of urban space and adapt to the growth of space and renewal of functions is an important task in urban planning, which would be useful to solve the contradiction between urban space growth and the situation of land resources [26]. Several studies are of great value for analyzing the development of rail transit stations and urban space. One study proposed a transportation system connecting independent areas with functional divisions such as commerce, residential, office, and leisure to guide public transport for neighborhood development [27,28]. Xu and Chen studied the spatial vitality and spatial environment of urban underground space (UUS) in a metro area based on spatiotemporal analysis [29]. Iseki et al. developed a time-of-day origin–destination direct transit demand model (OD-DTDM) that uses fare-card data from the Washington, DC, Metrorail system [30]. Wu et al. established a calculation model of station spacing for optimizing urban rail transit station space [31]. These are all effective ways to solve a series of problems involving cities, urban space, and urban traffic. 

### 2.3. Study of Transit-Oriented Development (TOD)

**Figure 1:**
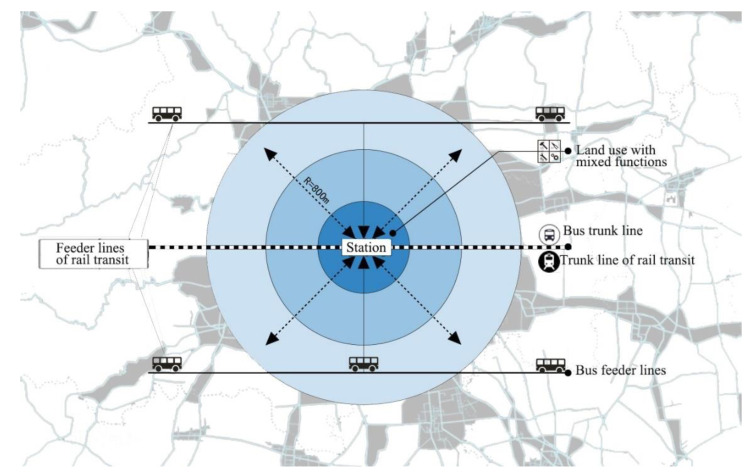
Transit-oriented development mode of city.

With increased traffic demand and changing urban spatial structure, the transit-oriented development (TOD) model is worthy of attention [32]. TOD is a method of non-motorized planning and design to maximize the use of public transportation. Public transportation mainly refers to railways, subways, light rail, other rail transit and bus trunk lines [33]. The city center is built with stations in a radius of 0–400 or 400~800 m (a 5–10 min walk), characterized as “mixed use”, integrating work, commerce, residence, culture, education, etc., that is, a “transportation hub + complex” [34], to achieve the organic coordination of compact development of each cluster, improve traffic congestion [35], achieve efficient utilization of resources [36], and create new economic growth. Residents and employees can easily choose public transportation, bicycles, walking, or other travel modes without excluding cars. Building on urban reconstruction plots, filled plots, and newly developed land can be done based on the TOD concept. Figure 1 is reproduced from a Baidu TOD picture, which indicates that the main function of TOD is to resolve the contradiction between traffic congestion and land shortage during urban development through land use and transportation policies [37]. Using this mode can realize and accelerate the process of city integration through transportation hubs, commercial functions, and urban planning (Figure 1).

In the early 1990s, based on reflections of suburban sprawl, a new urban design movement, neotraditional planning, emerged in the United States, which later evolved into the more well-known New Urbanism. The concept of TOD was first proposed by Peter Calthorpe, a practitioner of New Urbanism, in 1992. The development model of suburban sprawl was replaced by TOD, and detailed guidelines were formulated for land use based on TOD strategies. The purpose was to address the unrestricted spread of American cities after World War II and adopt pedestrian-friendly urban areas with public transport as the center and comprehensive development [38]. Countries that developed the TOD model include the United States, Japan, and Denmark. According to research at the University of California in 2002, as many as 137 transit-oriented development projects in the United States had been completed, were under development, or were being planned. Around the world, with the mode of car travel becoming predominant, cities have experienced large-scale spatial expansion with suburban sprawl. This has led to the migration of urban populations to the suburbs, the reduction of land use density and the decentralization of urban density. As a result, the decline of urban centers, the rupture of community ties, and environmental problems [39] have increasingly attracted social attention.

At present, the TOD model can be divided into urban and regional TOD [40]. Urban TOD refers to mixed development areas centered on metro, light rail, and other large-volume public transportation dominated by residential, public service, and commercial functions. Regional TOD refers to high-intensity mixed development areas with high-speed railways and intercity railway stations as the center, and commercial and business functions playing a leading role. This type of TOD will eventually form sub-centers or urban business centers. The essence of TOD mode is that urban public transport serves people’s travel activities and traffic brings passenger flow [41]; crowds are closely related to urban functions, urban functions provide a material basis for crowd activities, and crowd activities reflect the functional value of urban communities. TOD mode has become the most representative urban commercial development mode in the world and the next growth point of urban development [42,43]. The application of TOD will further enhance the value of cities based on the land development model of transportation-service facilities-land use and can effectively guide the population toward station centers and provide an important basis for land use and urban planning. We selected the world literature on rail transit and urban succession which are shown in Table 1.

## 3. Research Design

### 3.1. Research Objective

The land use around rail transit stations can represent the development status of commerce, residence, industry, entertainment, etc., and the development of a city will form station zones driven by rail transit, which include land use, building use, number of passengers, land price, and the scope of impact on the area. Both people’s lives and the land and building use inside and outside station zones can be affected. Hence, empirical study shows that promoting the development of station zones and the formation of dense urban areas, and further planning and rectifying the situation of land and building use around station zones are critical to improving the convenience of railway and subway stations. Hence, this study analyzed the urban development around train and subway stations in the city of Fukuoka over the last 30 years, in order to understand the mutual relationship between transportation infrastructure and land use. We chose the city of Fukuoka, Japan as a case study in view of its unique geographical and transportation advantages.

**Figure 2:**
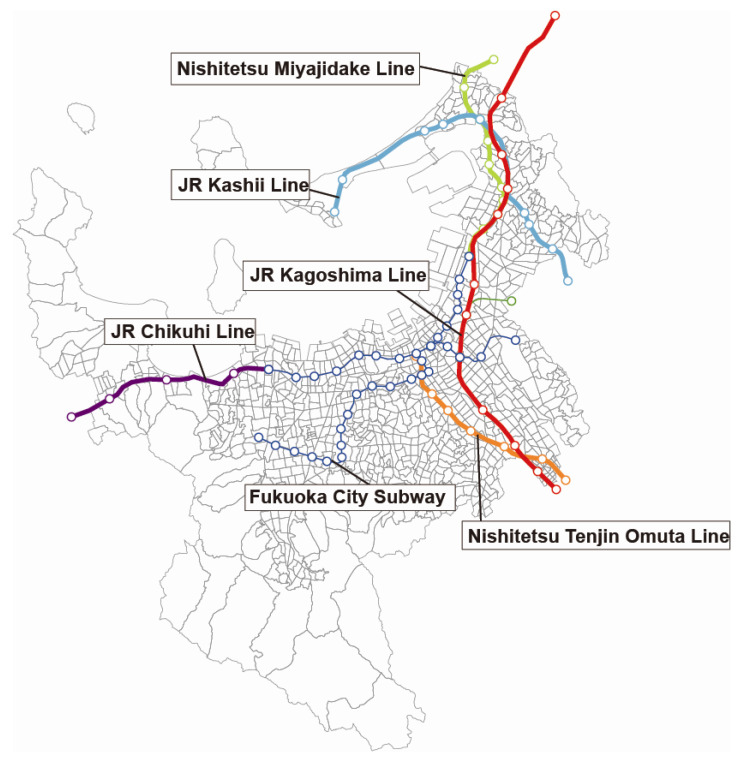
Overview of rail transit lines in Fukuoka, Japan.

Fukuoka, the sixth largest city in Japan, is located in the north of Kyushu. It is an important international metropolis and is the home of Fukuoka Prefectural International Hall. Fukuoka has a special geographical location, close to major cities of Japan (Osaka, Tokyo, Sapporo) and of East Asia (Busan, Seoul, Shanghai, Beijing). Besides the domestic transportation routes, there are also many regular international routes, making it the best city to communicate with Asian countries. Japan Railways (JR) is a large railway company group. Its predecessor is Japan’s state-owned railway (the National Railway, commonly abbreviated as JNR). In 1987, the National Railway of Japan was divided into seven companies and the original state-owned management rights were transferred to the private sector (i.e., it was divided and privatized). The separate companies are collectively called the Japan Railway Corporation, which is sometimes translated as Japan Passenger Railway, but it also includes freight railway companies. Thus, it is also called the Japan Railway Corporation Group (JR Group). This study takes rail transit stations of Fukuoka as the research object, including three belonging to Japan Railways, the JR Kagoshima Line, the JR Kashii Line, and the JR Chikuhi Line; two belonging to West Japan Railways (Nishitetsu Railway), the Nishitetsu Miyajidake Line and Nishitetsu Tenjin Omuta Line; and the Fukuoka Subway, totaling 68 rail transit stations (Figure 2). 

### 3.2. Data Collection

The detailed data used in this study mainly included land and building use (from POSMAP data of 6 periods at 68 stations in 1985, 1993, 1998, 2003, and 2008), geographic information system (GIS) data extracting land and building use around each station, and data of population and households from the population census of Fukuoka. The unit for population and household data is “chome” (a special street unit in Japan; in Japanese, “cho” is a street unit and “me” means the number, so 1-chome represents the first block). There are about 1100 chome in Fukuoka, which has increased in recent years with the change of addresses. In addition, we referred to the Fukuoka City Statistics Book for the number of passengers on Japan Railways and Western Japan Railways from 1975 to 2013, and the data of Fukuoka Subway from 1981, the opening year, to 2011. From the homepage of the Ministry of Land, Infrastructure, Transportation and Tourism, we collected data from the survey on land prices in the Digital National Land Information of Japan from 1983 to 2013. The architecture data include building confirmation in Fukuoka from 1985 to 2013, totaling 28 years. Finally, the data were integrated through GIS. Since the metropolitan areas around rail transit stations in Japan have stabilized and changed little since 2012, this study mainly focused on the period 1985 to 2010, including three succession stages spanning almost 30 years. This was also the golden period of urban development, transition, and finalization, which had a far-reaching impact on urban construction and rapid development of urbanization after the war. 

Data on land and building use around Fukuoka railway and subway stations and POSMAP were extracted from ArcGIS as the database. Based on the statistical yearbook of Fukuoka City, the average passengers per day and population were obtained. According to the land use map, detailed residential map, base map of building application registration, and urban planning map, the data of land use and street views of Fukuoka were obtained. We further analyzed the succession of existing rail transit station zones, as well as the development and utilization of surrounding areas and the urban environment, so as to find the transition trends, distribution, and changes from 1985 to 2010, then obtain the transition characteristics of station zones in Fukuoka through the typology method. 

### 3.3. Overview of Target Lines and Stations

JR Kyushu Kagoshima Line connects cities in northeast and the south, and Nishitetsu Tenjin Omuta Line connects cities in the south. These two lines cover the main transportation networks among city clusters. The Fukuoka Subway Airport Line and Hakozaki Line connect Meinohama–Tenjin–Fukuoka Airport with Kaizuka, linking up the city center from east to west and connecting with the JR Kyushu Kagoshima Line in Hakata by the Nishitetsu Tenjin Omuta Line, while the Nanakuma Line connects to the southwest of the city. In March 1994, the subway airport line was extended to Fukuoka Airport, and the laying of dual tracks was completed in January 2001. In February 2007, Nanakuma Line was opened. All these projects were implemented to enhance and improve the traffic network (Table 2).

(1) JR Kyushu Railway.

The JR Kyushu Railway has three main lines running in Fukuoka, which support the transportation networks: 

JR Kagoshima Line (Kaizuka crossing to Myoken crossing, 3490 m), which was transferred to Higashi Park by the prefecture government in 1981, includes road improvement around Yoshizuka Station; the government is always promoting the development of urban facilities in this district. 

JR Kashii Line: Hakata Bay Station was the predecessor of the Kashii Line, and its remnants have almost disappeared; recently, the Saitozaki transport for tourism, transportation, and delivery in Umi is the same routine.

JR Chikuhi Line: The western terminal station connecting JR Kyushu Chikuhi Line and Fukuoka Subway stations directly in the center of Meinohama Station, which was started in 1978 and finished in 1983. The data show that there has been obvious growth of estates around Meinohama Station, and the population is significantly higher. In order to ensure a good livable environment, land readjustment is under way to the south and west of Meinohama Station. The JR Kagoshima Line plays an important role in linking Fukuoka and metropolitan areas, which includes many elevated platforms and land readjustment projects around stations.

(2) Nishi–Nippon Railway.

There are two Nishi–Nippon Railway Lines in Fukuoka: the Tenjin Omuta Line, which connects Kurume City and Omuta City, and the Kaizuka Line, which connects the eastern and northern suburbs and Shingu Street. Since 2007, stations beyond Nishitetsu Shingu, the line that connects Fukuma Station and Tsuyazaki Station, were stopped because of fewer passengers.

The Tenjin Omuta Line was opened in 1924, and at that time, new stations in Fukuoka were not set. The elevated railway and land readjustment projects in Ohashi Station and Takamiya Station were carried out actively, so that Ohashi Station was positioned as a sub-center of Fukuoka. In addition, in the Kashi area, Nishitetsu Kashii Station, Kashiimiyamae Station, and Nakano Station (now Nishitetsu Chihaya Station) became elevated stations and Meinoshima Station was reconstructed. The Chihaya area gradually became a sub-center of the eastern district due to large-scale land readjustment.

(3) Fukuoka Subway.

There are three subway lines in Fukuoka. With the construction of subways, the capacity for transportation improved greatly and subways have become an important land vehicle in people’s lives. In addition, for more convenient access to the city center, exploring the downtown prevails. The Airport Line is a major route on the subway lines, and includes Hakata and Tenjin, thriving commerce and business centers around the Gion and Nakasu Kawabata areas. The Airport Line also stretches to the western suburban residential district. Due to the regression phenomenon of the city center in recent years, subways have become more and more important.

There are mainly government agencies, educational institutions, welfare facilities, and hospitals around the Hakozaki Subway Line, including the Kyushu Prefecture Hall and the Osaki campus of Kyushu University. Kaizuka Station, the connection between the West Railway and Kaizuka Line, has more educational institutions because the surrounding facilities are mostly government agencies, schools, hospitals, etc. The Nanakuma Line was opened in 2005, bringing great convenience to the residential areas in the southwest of Fukuoka. However, due to the smaller number of passengers, development around the station is not active compared to other stations. In addition, transportation from Tenjin Station to Tenjin Minami Station is not very convenient. The future plan is to extend the subway line and improve land development around the Nanakuma Line.

Furthermore, due to redevelopment after 1990, land planning readjustment projects were carried out. Since then, many places around stations have been redeveloped and land planning readjustment projects have been carried out. Since the late 1980s, many projects related to railways has been developed, such as the setting of new stations, the opening of the Airport Line and Hakozaki Line and the Nanakuma Subway Line, land readjustment projects, and urban street redevelopment. These projects were connected with the railway networks of Fukuoka, which greatly changed the city.

## 4. Research Methodology

### 4.1. Setting of Station Zones

This study mainly focuses on land use changes around each station and a comparison of distance. The most convenient method for this study was to set station zones. There are about 1100 chomes in Fukuoka. In recent years, that number has increased as the addresses have changed. Geographic information system (GIS) was used to integrate the data. GIS is a “computer-based system for the integration and analysis of geographic data”, part of a “larger constellation of computer technologies for processing geographical data”.

**Figure 3:**
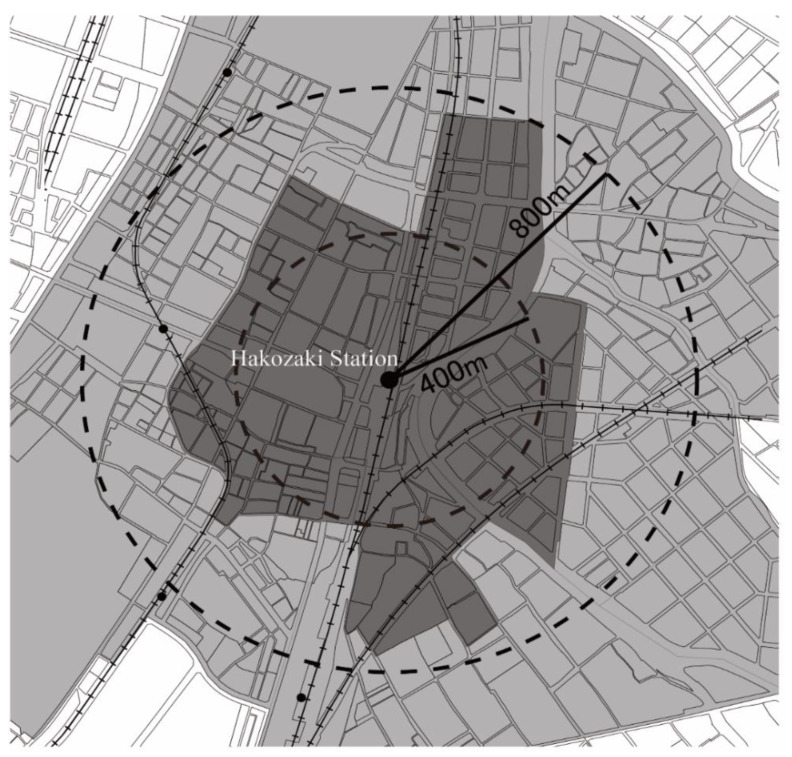
Setting of influence radius of station zones.

In January 2009, there were 74 stations in Fukuoka City (except the Shinkansen bullet train). The goal of this study was to analyze the transition of station zones; thus, circles with 0–400 and 0–800 m radius from the center point of each station were set, in relation to the spatial location of chomes. Figure 3 shows the setting of Hakozaki Station, a main subway station in Fukuoka, as an example. The dark grey represents the overlapping chomes of 0–400 m radius from center of object station, and the dark + light grey represents the overlapping chomes of 400–800 m radius from center of object station.

### 4.2. Research Methodology

This study focused on the urban spatial succession and transition characteristics of Fukuoka rail transit. GIS was used as a tool to extract POSMAP data from the location maps of land and building use around station zones by year, usage, and distance. 

There were 13 variables related to land use: commerce, house, government and education, industry, transportation, park and green land, agriculture, forest, road, water, developed open space, undeveloped open space, and others. After factor and cluster analysis of the variables, characteristics of different groups are discussed according to the distribution of land use in each group.

The characteristics of different types of stations in the same period and the changes in characteristics of the same type of station in different periods were clustered in order to clarify the development trend of station zones in Japan. The data of each station were analyzed in five-year stages. The analysis mainly included two steps to form station zones: taking a station as the center of a circle, within walking distance of the station, step 1 is to set a radius range of 0–400 m (a 5 min walk) and step 2 is to set a radius range of 400–800 m (a 10 min walk).

We used factor analysis to choose variables from the original data; the common factors in factor analysis are those that cannot be directly observed but exist objectively. Each variable can be expressed as the sum of the linear function of the common factor and a special factor:

(1) $$X_{\dot{i}}=a_{i1}F_{1}+a_{i2}F_{2}+\ldots +a_{im}F_{m}+\varepsilon_{i},\;\left(i=1,2,\ldots ,p\right)$$

where $F_{1}$, $F_{2}$, *…*, $F_{m}$ are the common factors and $\varepsilon_{i}$ is the special factor of $X_{\dot{i}}$. 

The model can be expressed as a matrix:

(2) X=AF+ε

where

(3) $$X=\left[\begin{array}{c} x_{1}\\ x_{2}\\ \vdots \\ x_{p} \end{array}\right],A=\left[\begin{array}{c} a_{11}\;a_{12\;\;}\ldots \;a_{1m}\\ a_{21}\;a_{22\;\;}\ldots \;a_{2m}\\ \ldots \;\;\;\ldots \;\;\;\ldots \;\;\;\;\ldots \\ a_{p1}\;a_{p2\;\;}\ldots \;a_{pm} \end{array}\right],\;F=\left[\begin{array}{c} F_{1}\\ F_{2}\\ \vdots \\ F_{m} \end{array}\right],\varepsilon =\left[\begin{array}{c} \varepsilon_{1}\\ \varepsilon_{2}\\ \vdots \\ \varepsilon_{p} \end{array}\right]$$

Furthermore, we used the squared Euclidean distance and Ward’s minimum variance method to analyze the distance between survey points, and we defined the cluster parameters. Cluster analysis assigns object data to groups (called clusters), such that objects in the same cluster are more similar than objects in other clusters. In other words, cluster analysis is a method to classify objects and provide certain convenience for analysis. Ward’s minimum variance is a method to minimize the sum of squares of cluster data, which is a special case of objective function; the standard for selecting clusters at each step is based on the optimal value of the objective functions. Cluster analysis by Ward’s method was used to find changes in station zones and spatial characteristics and development trends of stations over the years in order to provide a basis for the development of rail transit stations and surrounding areas. 

## 5. Results

### 5.1. Transition of Rail Transit Station Zones

#### 5.1.1. Changes in Acreage and Population in Station Zones

From the 1980s to the 2010s, the station zones expanded rapidly. In 1995, about 30% of the area, with 55% of the population, formed station zones. In addition, due to the opening of the Nanakuma Subway Line, residential areas were added in the southwest of the city, so that the area of station zones expanded to 42% of the total urban area, with 72% of the population in 2010. The urban streets basically radiate out from the station zones (Table 3).

The population of Fukuoka increased from the 1980s to the 2010s and appeared to return to the city center. During the past 40 years, Fukuoka has gradually formed a developed transportation network, supported by railways, subways, numerous arterial and connecting roads. With the development of rail transit and urban infrastructure since 1985, residential zones have become bigger, which has driven the development of businesses and real estate, some people choose to live far from the central areas and business districts considering of ecological environment and escaping from hustle and bustle in the congested city. Hence, the urban density of station zones was 6160 person/km^2^ in 1985 rose to a peak of 7089 in 2005 and fell to 6812 (a density lower than in 1995) in 2010. 

**Figure 4:**
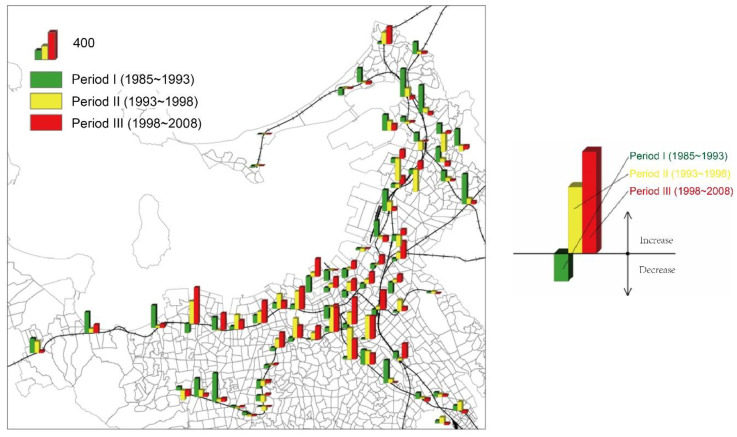
Population changes in station zones (average annual growth).

Furthermore, our study revealed that the number of passengers began to decline after a peak in 1996, but then increased again when the Nanakuma Line was opened. The investigation shows that passengers on JR lines increased dramatically after 1985 but decreased after the peak in 1996. The number of passengers on the Nishitetsu Railway Line was surpassed by those on JR Lines after a peak in 1991; after that, the difference gradually expanded. In addition, the Fukuoka Subway opened in 1981, and had up to 350,000 passengers in 1985, surpassing the other two lines and becoming the one with the most passengers, then the number further increased after the Nanakuma Line opened in 2006. It can be found that the number of passengers began to decline after the peak around 1996, and there was a trend of more people choosing residences far from the railways year by year, because cars are more convenient due to the urban highways and trunk roads around station zones, and the construction of shopping malls proceeded accordingly (Table 3, Figure 4).

Figure 4 shows the average annual population changes in station zones and the average annual growth rate within the influence range of stations in three periods: period I, 1985–1993; period II, 1993–1998; and period III, 1998–2008. In the figure, annual population growth in the three periods is indicated in green, yellow, and red, respectively. The vertical bar above the horizontal line represents the increment. It can be seen that population growth in the urban center basically occurred in the third period, and in the suburbs mostly occurred in the first period. Based on this, we can conclude that the population in Japanese rail transit station zones returned from the suburbs to the city center in recent years.

#### 5.1.2. Changes and Development Trends in Land Use around Station Zones

**Figure 5:**
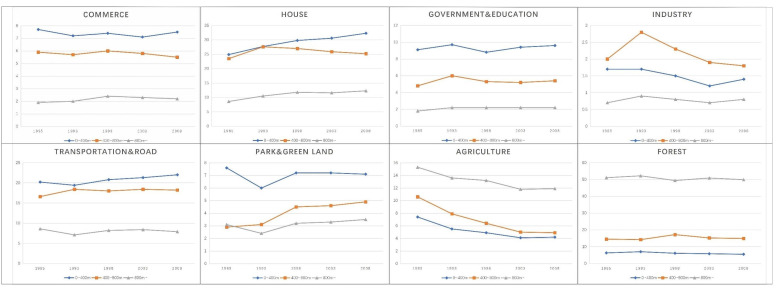
Changes in land use around station zones (%).

Figure 5 shows the proportion of land use within the influence area of station zones from 1985 to 2008. From 1985 to 2010, the proportion of residents increased dramatically, and land tended to be developed for residential use. Meanwhile, the proportion of land used for agriculture and forest declined. Compared with the radius of 400–800 m, the closer to the station, the higher the proportion of commercial land, and the proportion of residential land rises sharply. In addition, land use within a radius of 0–400 m of a station zone includes a high proportion of government, education facilities, and parks and green spaces. The facility configuration in this district is more complete than that within the 400–800 m radius outside the station zone. In the urbanization control area outside the station zone (800+ m), the proportions of agricultural land and forest land are relatively high, but the proportion of planned land relative to agricultural land declined and the developed agricultural land was used for residential and commercial land.

From the comparison of the development span of land use around station zones over the 30 years under study, it can also be found that park and green spaces in the central area of Fukuoka gradually increased. The proportion of forest continually increased in the 0–800 m and 800+ m radius from 1985 to 2008, and the proportion of residential buildings increased in both. Comparing the data of 0–400 m, 400–800 m, and 800+ m radius, the proportion of commercial land declined somewhat, the ratio of industry was reduced, the proportion of agriculture land in the suburbs also tended to decrease, and government and education land also declined from the 0–400 m radius to the 800+ m radius, which can be seen from the ratio of land use of station zones (Figure 5). 

#### 5.1.3. Changes and Development Trends in Building Use around Station Zones

From 1985 to 2010, according to the proportion of detached houses and apartments, detached houses gradually decreased, and apartments tended to increase, which can be seen in the distance of all segments. It is also noticeable that detached houses were reduced by half and apartments doubled from the center of the station zone to the 400 m range. However, in the 400–800 m range and outside the 800 m range, the situation was different. The change trend of detached houses and apartments in the 400–800 m range and outside 800 m was slow. In the range of station zones, there was a short reverse bias in the proportion of detached houses and apartments, whereas outside the station zones, there was still a high proportion of detached houses. 

Commerce, business facilities, government agencies, and education facilities are highly distributed in the center of stations, not only around residential areas, but also with various other buildings for different uses. Transportation and storage facilities were distributed within a radius of 400–800 m and tended to be slightly away from the center of the station. In addition, a change trend of plot ratio can be seen within 400 m from the center of the station, indicating that the surrounding area was under development. Within the radius of 0–400 m from the center of the station, the population density shows a rapid growth trend. At the same time, it can be seen that the proportion of condominium increased (Table 4).

Table 4 also shows that urban density in the second zone around the stations dropped significantly from its peak in 1993 by almost 40%. This despite a rise in condominiums. with the development of urbanization and the increase of redevelopment projects around the stations, residential zones have become bigger, the change trend of condominiums was increasing, however, as the acceleration of the aging process and declining birthrate in Japan, the family size and population density tend to be reducing.

#### 5.1.4. Changes and Development Trends Inside and Outside Station Zones

Table 5 analyzes the amount of development of buildings and the distribution changes in their use around stations. With regard to development volume around station zones, there was a decreasing trend from 1996. In addition, it can be seen that in Japan, the proportion of apartments in terms of building use increased year by year. With a decreased proportion of independent houses and increased proportion of affordable houses, the distance from the station center also gradually extended, and the change was slower and slower. With regard to the number of shops and restaurants, between the station center and a radius of 400 m they increased to a certain extent, and the distance between shops was reduced. In addition, from the center of the station to the radius of 400 m, the proportion of office space had an increasing trend, but there was still a slight decline, similar to the changes at 400–800 m in 2002. Table 5 shows the changes in development trends of station zones.

Through the above analysis, it can be seen that, compared with the population growth trend in Fukuoka City, Japan, the number of rail passengers shows a downward trend. In addition, the development centers inside and outside station zones were transferred to the inner circle of stations, the proportion of independent houses declined, and the proportion of apartment buildings and rental houses increased. Especially in the areas surrounding the 0–400 m radius of the stations, the change trend of buildings was more obvious, reflecting the phenomenon of increased construction of high-end houses. The number of passengers shows a downward trend on the whole, but station reconstruction, lifting, and projects related to land consolidation increased. The number of passengers also shows an increasing trend after the completion of these reconstruction projects. On the contrary, stations that did not undergo reconstruction and rectification continued to show a decrease in passenger volume year by year. Therefore, station reconstruction and rectification projects are of great significance to increase the passenger volume and promote the development of surrounding areas. 

**Figure 6:**
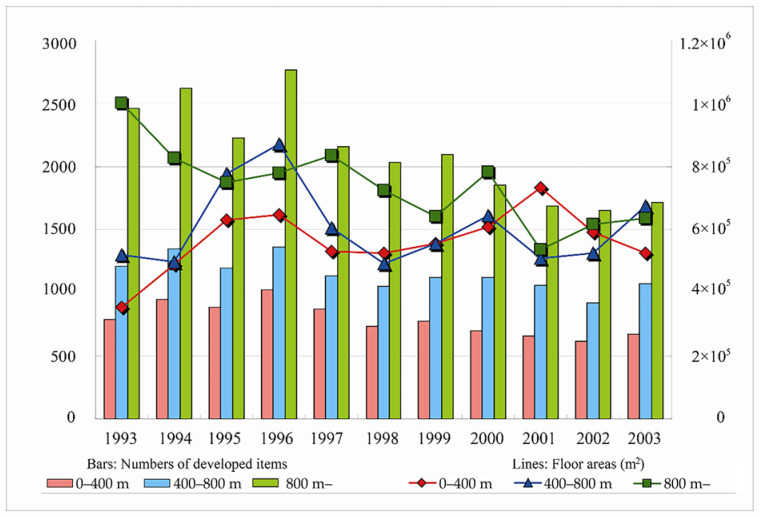
Amount of development areas around rail transit stations.

Regarding the proportion of inner and outer station development areas, compared with the proportion of outer station development of about 45% in 1995, it can be seen that the proportion decreased in 2003, and the development within a 400 m radius around the station increased, then the proportion of outer station development was 35%, and the proportion within a 400 m radius was also 35%. We can see that the development center transferred to areas within a radius of 400 m around the stations in recent years (Figure 6, Table 6).

The numbers of developed items of three zones kept a stable increase since 1993. Figure 6 also revealed the rapid development period during 1993–1996, which reached the peak in 1994 and 1996 and had lost since then, but there was still continuous development after 1997 in three zones. From annual change rate of development areas around station zones, it can be seen that a 400 m radius had an obvious change since 2002 and reached a peak in 2008, while change rate in 400–800 m radius reached 37.9% in 1996. Change rate of station zones of 800+ m range remained above 30% with a peak of 45.5% in 1994, back to 33.9% in 1996 which indicated the impact of Japan’s bubble economy. 

Analysis on transition of rail transit station zones from 1985 to 2010 has shown how transportation and land use interacted over time, how building use and development amount changed around station zones, what characteristics represented at different succession stages, especially changes in the golden period of urban development. The results also tell us, after the Second World War, urban development in Japan recovered and grew rapidly; after Japan’s bubble economy, urban construction and transportation development are still in process which have entered a relatively slower but steady stage. 

### 5.2. Results of Factor Analysis

**Figure 7:**
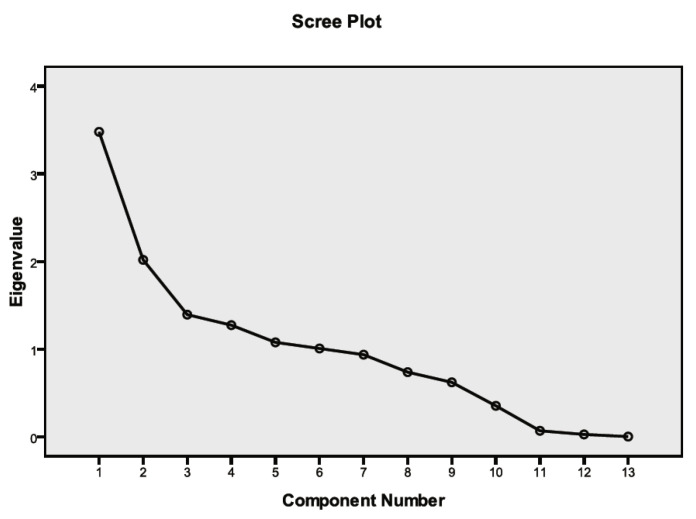
Scree plot showing characteristics of each component.

As mentioned above, there are 13 variables related to land use: commerce, house, government and education, industry, transportation, park and green land, agriculture, forest, road, water, developed open space, undeveloped open space, and others. These variables were extracted from the original data by factor analysis. We extracted common factors from variable groups and found hidden representative factors in the variables. We can reduce the number of variables and test the hypothesis of the relationship between variables by grouping variables of the same nature into a factor. Before using principal component analysis to extract the 13 original variables, all initial values were set to 1.000. In Table 7, extraction indicates the proportion of variance of each variable that can be explained by the factors. Eigenvalues indicate the variances of factors. The first six principal components with eigenvalues >1 were extracted. Variance extraction analysis shows that the cumulative variance contribution of the first six components accounts for 78.844%, which means that it can explain 78.844% of the information of the 13 original variables (Table 7). Figure 7 is the scree plot showing characteristics of each component. Table 8 represents component transformation matrix. 

In this study, we used principal component analysis to extract variables and sort out the factors with very small influence on others (water, developed open space, undeveloped open space), and closely related variables fell into the same category, so each type of variable became a factor. A few factors can reflect most of the information of the original data.

The influential variable of factor 1 (F1) is commerce, which includes the city’s entertainment, businesses, restaurants, shops, etc., and represents the level of commercial development of the city. Thus, F1 is named the commerce factor. Factor 2 (F2) is more related to the number of residents; this cluster is named the house factor and represents residential density and empty spaces. Factor 3 (F3) mainly consists of government, schools (middle schools, high schools, universities, educational institutions), and other public service facilities. F3 is public management and service. Factor 4 (F4) has higher loadings in park and open space areas, mainly linking the variables of parks, green land, forests, etc., so it is the park and green land factor. Factor 5 (F5) mainly reflects the development of traffic and roads, so it is the transportation and road factor. Factor 6 (F6) represents other variables including industry, agriculture, undeveloped open space, and others, and due to the large proportion of industry, it is the industry and others factor (Table 8). 

### 5.3. Results of Cluster Analysis

As described in the previous overview, the Nanakuma Subway Line was opened in 2005, and its characteristics and transitions have not yet been fully reflected; thus, we removed this subway line from cluster analysis, and 52 stations remained. Based on the population data, the number of passengers and land use of 52 stations in 5 years (1985, 1993, 1998, 2003, and 2008), we used the above data to perform cluster analysis. Cluster analysis is a technique that directly compares the properties of various things, and it classifies those with similar properties into one category and those with large differences into other categories. The factor analysis extracted 6 variables from 13 variables and made similar ones into one variable in order to form a more reasonable base for the cluster analysis: commerce, house, public management and service facilities, park and green land, transportation and roads, and industry and others. Then, data of chomes in 0–400 m and 400–800 m buffer zones around the centers of stations and the configuration ratio of land use were aggregated. 

#### 5.3.1. Typology and Characteristics of Rail Transit Stations

In order to explore the spatial characteristics of different types of rail transit stations, the area proportion of land use, population, and passengers per day in 0–400 m and 400–800 m buffer zones were added as cluster factors, which are factors that influence station characteristics. The derivative square and Euclidean distance square were used to decide the distance, and the 52 stations were divided into 6 clusters, so we could analyze the urban spatial scale, characteristics, and changes of each station. 

Based on the statistical yearbook of Fukuoka City, the average passengers per day and population were obtained. According to the land use and urban planning maps, the data of land use of Fukuoka were obtained and classified into commercial land, residential land, public management and service facilities, park and green land, transportation and roads, and industry and others. According to the results of cluster analysis, the characteristics of spatial succession of each station were obtained, and the classifications for 0–400 m and 400–800 m are given in Table 9, Table 10, Table 11 and Table 12.

- Step 1. 0–400 m radius.

The characteristics of each group in 0–400 m radius can be summarized as follows (see Table 10):

(1) Group 1: Commerce (11 stations).

In this group, commerce accounts for the highest proportion, 27.9%, which represents the highest average among all groups; the average passenger flow per day and population proportion are also relatively high. The proportion of residential areas is the lowest, because many stations are concentrated in areas surrounding the city centers, where there are many residential areas. People get accustomed to shopping and having entertainment in this group, but they choose to live in other places. Therefore, stations in this group gradually became commercial-centered. Furthermore, it is obviously shown that the percentage of park and green land is very low, but transportation and roads in group 1 accounts for the highest percentage among all the groups, at 22.9%.

(2) Group 2: Public management and service (8 stations).

In this group, the proportion of commerce is second only to group 1, and the ratio of public management and service facilities is up to 25.2%, which is considerably higher than the other groups; the proportions of municipal institutions, schools, and other education facilities are also much higher than the other groups, and the proportion of residential areas is higher. Moreover, the percentage of commerce in this group is much lower than group 1, which shows that stations in this group are tending to become commercial-centered, and this group will become a sub-center of the first group in the future. The percentage of housing (32.7%) in this group is similar to group 5, with low-density residence and industry (31.2%) in the form of a few houses built around public management and service facilities. In addition, from the data analysis, it can be seen that the proportions of park and green land and transportation and roads in this group are higher.

(3) Group 3: High-density residence (13 stations).

In Japan, with land consolidation projects and construction along railways and subways, rail transit is changing rapidly, and the residential areas of cities have gradually shifted to the suburbs. The density of housing surrounding rail transit stations is increasing year by year, the development trend in commercial zones around stations is toward diffusion, and urban planning is gradually placing parks and green land away from the center of station zones. Meanwhile, with the development of large-scale retail stores, the closure of commercial streets has become increasingly apparent. There are many commercial streets in Japan, almost covering the cities, composed of many small shops. They used to be bustling shopping areas, but now these shops are closing, leaving most of these areas as residential. 

The proportion of residential areas in this group is the highest compared to other groups, at 56.1%. Clearly the variable housing plays an important role in this group, which is located in high-density residential areas and also frequently around railways and subways. The proportion of park and green land in this group is higher than in other groups. Commerce in this group also accounts for a lower proportion, at 5.6%. According to the field survey, some sites of this group are developing commerce gradually. Furthermore, the ratio of public management and service facilities of this group is less than other groups, at only 3.9% in average.

(4) Group 4: Medium-density residence (10 stations).

The percentage of housing in this group is the second highest among all the groups. There is a higher population ratio in this group, due to the higher density of residential areas. However, the percentage of park and green land is very low in this group, at only 2.8%. The proportions of transportation and roads and industry and others are in the middle among the six groups, at 19.1% and 17.3%, respectively. Some station zones, such as the Yoshitsuka station zone, are developing faster than before, not only in commerce, but also in transportation and roads. The proportion of public management and service facilities in this group is much higher, therefore, it can be considered that land use by municipal institutions, schools, and other educational facilities established around the stations in this group is relatively high. In addition, the percentage of commerce is far lower than that of commercial stations in group 1.

(5) Group 5: Low-density residence (6 stations).

Among all stations, the percentage of housing in this group is up to 31.2%, which is lower than groups 3 and 4. By using a mesh for population with GIS, it is shown that the population in this group is the lowest among all the groups. The proportion of park and green land is very low; it was 2.7% in 1985 and only 2.4% until 2008. It can be seen that the construction of park and green land around these station zones has declined in the past 23 years. The proportion of transportation and roads is the lowest among the six groups; it was 13% in 1985 and about 17% in 2008. Notably, industry and others account for the highest percentage among all groups, at 40%. According to the fieldwork, stations and land use in this group have always had various other functions, such as trade communication, industry, warehousing, etc. There is also much unused land that will be considered in planning programs in the future.

(6) Group 6: Park and green land (4 stations).

The proportion of park and green land in this group is up to 17.4%, the highest among all groups. The amount of green land around stations of this group is beneficial for sustainable development in urban modernization. Furthermore, the number of passengers per day is also the least among all groups, and the industry and others category is second only to the other category in group 5. The average proportion of housing in this group is lower (28.6%).

- Step 2. 400–800 m radius.

The results of cluster analysis were divided into six groups (see Table 11). The characteristics of each group in the 400–800 m radius can be summarized as follows (see Table 12):

**Table 11:** Station classification in 400–800 m radius after cluster analysis.

Type	Station
Group 1: Commerce	Nishitetsu Fukuoka Tenjin, Akasaka, Tenjin, Nakasu-kawabata, Gion, Hakata, Higashi-hie, Gofukumachi
Group 2: Public management, service, and low-density residence	Ohashi, Kaizuka, Fujisaki, Chiyo-kenchoguchi, Maidashi-kyudaibyoinmae, Hakozaki-kyudaimae
Group 3: High-density residence	Chikuzen Shingu, Sasabaru, Susenji, Kashiijingu, Maimatsubara, Nishitetsu Kashiikaenmae, Mitoma, Muromi, Nishijin
Group 4: Medium-density residence	Kyusansai-mae, Kashii, Chihaya, Takeshita, Minami-Fukuoka, Meinohama, Shimoyamato, Yakuin, Nishitetsu Chihaya, Kashiimiyamae, Nishitetsu Kashii, Tonoharu, Hakozaki-miyamae
Group 5: Industry and others	Hakozaki, Yoshizuka, Imajuko, Nata, Wajiro, Doi, Zassyonokuma
Group 6: Park and green land and transportation	Saitozaki, Gannosu, Najima, Tojinmachi, Ohorikoen, Fukuokakuko

**Table 12:** Result of cluster analysis of 400–800 m radius (including land use, population, and passengers).

Cluster	Station	Line	Land Use						Passenger (Person/ Day)	Population (Person)
			Commerce	House	Public Management & Service	Park & Green Land	Transporta-tion & Road	Industry & Others		
			(%)							
	**Type of Commerce**									
	Nishitetsu Fukuoka Tenjin	Railway	40.8	32.7	8.2	6.9	10.9	0.5	50,188	17,652
	Akasaka	Subway	30.3	36.0	18.7	10.8	3.1	1.1	9628	22,808
	Tenjin	Subway	42.9	29.7	9.9	6.4	10.4	0.8	43,591	19,468
	Nakasu-kawabata	Subway	38.9	25.6	12.5	11.3	11.0	0.7	9353	19,494
8	Gion	Subway	31.6	23.6	17.6	10.1	16.0	1.2	4431	20,025
	Hakata	Subway	39.7	28.7	5.5	8.7	14.1	3.3	39,699	23,238
	Higashi-hie	Subway	36.2	21.7	2.5	14.3	13.4	11.9	5663	12,992
	Gofukumachi	Subway	42.8	17.8	13.5	7.8	16.7	1.4	1986	19,008
	**Average**		37.9	27.0	11.0	9.5	11.9	2.6	20,567	19,336
	**Type of Public management, service and low-density residence**									
	Ohashi	Railway	5.2	50.9	19.2	9.0	12.2	3.5	12,596	19,941
	Kaizuka	Subway	10.9	20.0	42.7	6.5	12.5	7.5	4587	3736
	Fujisaki	Subway	6.2	56.2	24.7	5.9	5.5	1.5	7316	32,764
	Chiyo-kenchoguchi	Subway	27.2	27.0	22.4	12.3	9.3	1.9	2315	18,209
6	Maidashi-kyudaibyoinmae	Subway	14.9	36.6	35.2	6.0	3.4	4.0	2912	24,677
	Hakozaki-kyudaimae	Subway	9.9	30.8	36.2	7.8	11.4	3.9	2325	6055
	**Average**		12.4	36.9	30.1	7.9	9.0	3.7	4955	17,564
	**Type of High-density residence**									
	Chikuzen Shingu *	JR	7.6	58.5	4.8	8.7	10.7	9.8	8177	7711
	Sasabaru	JR	12.2	58.9	5.7	10.7	8.9	3.8	2694	22,606
	Susenji	JR	7.6	60.6	3.6	8.6	16.4	3.2	5653	8086
	Kashiijingu	JR	2.0	59.1	12.2	19.3	2.2	5.1	645	17,556
	Maimatsubara	JR	5.4	57.6	12.5	16.0	3.9	4.6	664	18,169
12	Nishitetsu Hirao	Railway	11.8	65.0	11.1	6.1	3.7	2.3	4348	36,633
	Takamiya	Railway	8.7	61.2	14.9	9.1	3.3	2.9	6843	17,853
	Ijiri	Railway	6.0	67.8	2.6	11.8	6.4	5.4	7986	22,273
	Nishitetsu Kashiikaenmae	Railway	5.8	59.7	8.0	14.2	5.8	6.6	967	10,755
	Mitoma	Railway	3.1	63.2	9.3	9.2	4.1	11.1	1184	9222
	Muromi	Subway	4.7	65.0	9.6	5.7	12.9	2.2	5154	25,941
	Nishijin	Subway	7.1	64.9	17.6	5.9	3.9	0.5	14,910	30,412
	**Average**		6.8	61.8	9.3	10.4	6.9	4.8	4935	18,935
	**Type of Medium-density residence**									
	Kyusandai-mae	JR	5.2	50.6	13.9	15.1	9.1	6.1	4549	13,351
	Kashii	JR	7.6	52.4	12.2	15.3	7.1	5.4	8493	20,938
	Chihaya	JR	8.2	54.5	7.0	18.8	9.8	1.7	5128	23,837
	Takeshita	JR	10.9	44.4	18.3	8.2	14.0	4.2	3696	19,724
	Minami-Fukuoka	JR	14.0	47.6	6.4	9.7	19.6	2.8	6113	15,353
13	Meinohama	JR	13.3	51.2	11.4	10.3	11.4	2.3	4259	31,255
	Shimoyamato	JR	5.9	46.4	5.2	4.9	10.4	27.1	1561	20,329
	Yakuin	Railway	26.3	47.3	9.7	8.7	6.3	1.8	12,678	36,454
	Nishitetsu Chihaya	Railway	9.4	53.9	8.9	17.0	9.3	1.5	1208	2051
	Kashiimiyamae	Railway	8.6	46.5	13.9	19.5	9.0	2.6	880	20,715
	Nishitetsu Kashii	Railway	16.3	45.0	5.2	2.2	16.4	14.9	1170	14,295
	Tonoharu	Railway	2.8	50.2	6.3	22.5	6.2	12.1	380	6852
	Hakozaki-miyamae	Subway	8.3	43.5	11.5	2.7	22.3	11.7	2210	11,168
	**Average**		10.5	48.7	10.0	11.9	11.6	7.2	4025	18,179
	**Type of Industry and others**									
	Hakozaki	JR	15.5	35.5	11.9	12.3	17.0	7.7	2992	27,880
	Yoshizuka	JR	16.6	38.9	14.2	1.7	7.3	21.3	7178	21,481
7	Imajuku	JR	4.3	36.1	2.1	21.3	5.5	30.8	3119	6019
	Nata	JR	6.0	39.4	19.4	10.3	3.6	21.3	875	4578
	Wajiro	JR	11.3	32.5	4.2	25.7	5.3	21.1	1110	16,458
	Doi	JR	18.1	35.8	1.5	10.2	4.7	29.8	802	12,493
	Zassyonokuma	JR	5.7	21.3	9.0	8.7	2.9	52.4	3430	13,203
	**Average**		11.1	34.2	8.9	12.9	6.6	26.3	2787	14,587
	**Type of Park, green land and transportation**									
	Saitozaki	JR	1.9	23.6	2.2	53.1	2.8	16.5	613	1180
6	Gannosu	JR	0.7	16.8	2.4	65.6	1.1	13.5	244	3771
	Najima	Railway	6.3	35.5	6.8	13.2	32.3	5.9	629	20,428
	Tojinmachi	Subway	5.8	47.2	11.1	22.9	12.7	0.3	6367	26,075
	Ohorikouen	Subway	10.3	29.4	19.7	20.0	19.1	1.5	5394	14,336
	Fukuokakuko (Airport)	Subway	1.2	6.1	0.3	3.8	83.3	5.3	14,343	3227
	**Average**		4.4	26.4	7.1	29.8	25.2	7.2	4598	11,503

* The name of Chikuzenshingu Station has been changed into Fukkodai-mae Station in 2008.

(1) Group 1: Commerce (8 stations).

This group has a relatively high proportion of commerce, 37.9%, which is the highest among all the groups. The average passengers per day and population also have the highest numbers, 20,567 and 19,336, respectively. Thus, not only do stations in this group have a large flow of passengers, but this is also the busiest section in Fukuoka, especially Tenjin Station and Hakata Station, where so many passengers go shopping, work, or travel that two rather big business zones have formed. As a result, business zones have become bigger, which has driven the development of businesses, office buildings, and real estate around the railways, and continues to be important in facilitating the surrounding regions’ growth and prosperity. The percentage of housing is lower than in other groups. It can be clearly seen in Table 12 that this group and group 6 have a smaller proportion of housing, which is higher in the other four groups; this shows that most people choose to live far from the central areas and business districts. The ratio of transportation and roads is a little higher than some but not all groups, and the percentage of roads in the 0–400 m radius is the highest among all groups, which shows the importance of transportation in urban development. The ratio of park and green land in the 0–400 m radius is not so high but not so low, at 9.5%. Notably, the proportion of industry and others in this group is the lowest among all groups, at only 2.6% on average. It was also found that there is less unused space, forest, and water than other regions.

(2) Group 2: Public management, service, and low-density residence (6 stations).

In this group, the percentage of land use for educational facilities is up to 30.1%, which is an especially high number among the six groups. In fact, there is a high ratio of middle schools, universities, and other educational and government institutions around stations in this group, such as Fukuoka City Hall and Kyushu University, one of the best universities in Japan and the best in the district. At this point, most of the Hakozaki campus moved to the Ito campus, which has driven the development of catering industry, including roadside cafes and bookstores, around subway stations. According to the field survey, there are many more municipal offices and education facilities than in other districts. Moreover, the percentage of commerce in this group is obviously much lower than in group 1. The ratio of housing in this group is 36.9%, which is similar to industry and others in group 5 (34.2%), and almost the same as the situation in 0–400 m radius. The percentage of industry and others in this group is second only to group 1, which has the lowest percentage, indicating a lower ratio of undeveloped land and forest, etc. The average number of passengers per day is not so high but not very low in this group and much lower than group 1.

(3) Group 3: High-density residence (12 stations).

The number of passengers (4935) and the proportion of commerce (6.8%) in this group are lower than in other groups, but the population is not low on average, but much higher than some groups. Housing plays a very important role in this group, with a proportion up to 61.8%. Stations of this group are all located in high-density residential areas, so these are residential districts with a high frequency of land use for housing. It was found in the field survey that people usually demand a high-quality dwelling environment in these areas. Consulates of other countries are mostly distributed around Nishijin Station. People often make annual pilgrimages to the shrine near Kashiijingu Station, which is surrounded by a clean and quiet living environment. The proportion of municipal and educational facilities in this group are a little less than the other four groups, except public management, service, and low-density residence in group 2, which is only 9.3% on average. Roads in this group have a lower average (9.0%) than the other four groups, except industry and others in group 5. The percentage of commerce in this group is also very low, but according to the field survey, as the need for housing grows, more shopping malls and restaurants are built. For example, around Susenji Station and Nishitetsu Kashiikaenmae, some new entertainment facilities opened to provide more convenient services for those who live near the stations. This is just one example; there are many newly opened facilities for shopping around these stations, so more people are willing to live there, and more people who have always lived there are not willing to move away, which is why the percentage of housing is the highest among all the groups.

(4) Group 4: Medium-density residence (13 stations).

The percentage of housing in this group is the second highest of all the groups, at 48.7%, which determines a relatively higher need for transportation and roads, similar to commerce in group 1. There is also a higher population in this group, because it is a relatively high-density residential area. The percentage of park and green land is a little high, reaching 11.9%. The proportions of transportation and roads, and industry and others are in the middle of the six groups, up to 11.6% and 7.2%, respectively. Some stations, such as Yakurin and Nishitetsu Chihaya stations, are gradually focusing on developing sub-central commercial areas. The percentage of commerce around Yakuin Station was up to 26.3%, which is the highest in this group. The percentage of government and education for Takeshita Station reached 18.3%, which is higher than the other stations in this group. The percentage of park and green land reached 22.5%, which is higher than the average of this group (11.9%). In the category transportation and roads, Minami-Fukuoka and Hakozaki-miyamae stations have higher percentages than the other stations, with 19.6% and 22.3%, respectively, which indicates fast-growing transportation to meet the demand in people’s lives. Furthermore, there is a high percentage of not only undeveloped land, but also forest and other land, around Shimoyamato Station, up to 27.1%.

(5) Group 5: Industry and others (7 stations).

The percentage of industry and others is higher than in other groups, up to 26.3%. The data from field investigation show that there is a certain amount of industry and undeveloped land around these stations, especially Imajuku and Zassyonokuma stations. Notably, the percentage of housing in this group is up to 34.2% which is lower than groups 3 and 4, but similar to group 2 (residential average 36.9%). Hence, it is also a low-density residential area and the number of passengers per day is the lowest among the six groups. The ratio of park and green land is 12.9%, the second highest among all the groups. Although the percentage of residential area is lower than groups 3 and 4, the ratio of industry and others is the highest among the six groups at 26.3%. These data also show that there is a high ratio of unused land and undeveloped land (i.e., forest) in this group. The population data abstracted from the GIS clearly indicates that the population in this group is the second lowest, next to group 6. According to the fieldwork, stations in this group are charged with various functions and there is much unused land there that will be developed in the future. In this group, commerce needs to be further developed in the areas surrounding Imajuku and Nata stations; educational facilities around Doi and Imajuku stations should be improved; and the urban environment around Yoshizuka Station, which has 1.7% park and green land, should be improved, such as by the construction of an ecological park.

(6) Group 6: Park and green land, and transportation (6 stations).

The percentages of the categories of commerce, housing, and municipal institutions and educational facilities in this group are the lowest among all the groups (4.4%, 26.4%, and 7.1%, respectively). The percentages of park and green land, and transportation and roads are the highest (29.8% and 25.2%, respectively). Notably, the percentage of park and green land around Ganosu Station is the highest in this group, at 65.6%, and the percentage of transportation and roads around Fukuokakuko Station is the highest, at 83.3%. The famous Ohori Park is located at Ohorikoen Station, in the western part of Fukuoka. The park, which is built around the Fukuoka City moat with Japanese gardens, is an ecological park that people like to visit. Cherry blossoms in Ohori Park, nearby Maizuru Park (in Fukuoka City), and West Park are very popular every spring, and the area is regarded as a cherry appreciation resort. Annual fireworks are also held there; thus, Ohorikoen and Fukuokakuko stations have a major transportation function in Fukuoka, indicating that stations have different functions and play different roles in urban development. Moreover, in this group, there is much green land, which is beneficial for sustainable and ecological development of the city.

Through the above analysis, several findings are revealed:

(1) In Japan, the proportion of residential buildings around government and education facilities tends to be lower, and the proportion of commercial buildings was higher in the past 30 years. Due to the re-planning and readjustment of land use, the traditional shopping streets are shrinking and gradually disappearing. More people are accustomed to go shopping and enjoy entertainment in areas in the commerce group, but they choose to live in other areas. The proportion of transportation is gradually rising, therefore, stations in this group are becoming commercial-centered;
(2) It can be seen that the residential distribution in station zones has changed significantly in recent years. Due to the contraction of land in Japan, high-density residential areas mainly consist of apartments and mansions, the proportion of medium-density residential buildings is increasing, the number of independent houses is declining, and the area of parks and green spaces around residential areas is increasing. The growth of the proportion of parks and green space in station zones has changed significantly. It is particularly noteworthy that the development trend of housing is changing from single-family houses to mansions or apartments. Meanwhile, the distribution of construction projects has transferred to city centers;
(3) The proportion of land used for transportation and storage facilities has also been rising. Industrial and agricultural land use has not increased significantly, and these use types are far away from residential areas. The proportion of industrial and agricultural land has not changed much in the past 30 years. Since 2010, industrial land around the railways in Japan has been decreasing, and green land has been increasing. It can be seen that the Japanese government attaches great importance to environmental protection and urban regeneration;
(4) The development of city centers is mainly concentrated in the southern section of the national highways, and the number of large-scale projects increased, especially in the areas surrounding Sumiyoshi district. Future planning is focused on maintaining the balance of overall infrastructure construction;
(5) Along the Airport Line and Omuda Line, railway and transportation trunk lines are undergoing a comprehensive transformation, and areas with business centers have become scattered. There are many infrastructure projects around stations, and the railway and highway networks cannot form an urban transportation network, which is one of the problems of urban development. In addition, more agriculture land is distributed along the Nanakuma Subway Line and there is still a certain amount of development space. Based on the use of the TOD model in the future, it will be necessary to carry out transformation and integration according to the development mode of commerce centers;
(6) The urban form of high-density development makes urban traffic highly concentrated. From the fieldwork, we find that as Japan is an island country, rail transit is the most important mode of transportation, and it is also one of the few profitable urban railway systems in the world. Commerce, entertainment centers, and office buildings are concentrated within a few kilometers of railway stations, and there are street-level and underground passages to protect passengers from cars and harsh weather. As many activities are directly carried out near stations, rail transit is the most convenient and commonly used mode of transportation for people to get into and out of cities. There are typically a series of TOD communities radiating outward from suburban railways, and large community centers are arranged around the stations. There is a walking system from the center to nearby residential areas. It is convenient for residents to walk or take a bus to the railway station. The research shows that in Japan, 68% of residents’ trips to railway stations are on foot, 24% by bus, and only 6% by private car. Obviously, this land use configuration not only attracts long-distance travelers to use the railways, but also effectively reduces the volume of motor vehicle traffic within the community, thus alleviating problems such as the decline of urban centers, the rupture of community ties, and energy and environmental issues.

#### 5.3.2. Typological Maps of Land Use in Station Zones

**Figure 8:**
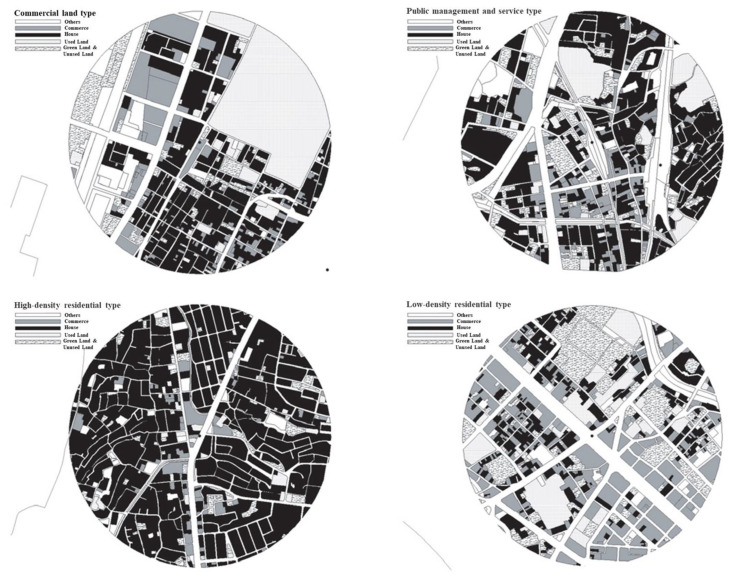
Typological maps of land use in station zones.

Based on the detailed theory proof, images, and data analysis, we can intuitively observe the differences in characteristics between classifications, and the typical land use can be illustrated as shown in Figure 8.

Figure 8 presents the typology of land use in station zones, which can provide clearer classifications of land use, including Gion Station (commerce), Saitozaki Station (public management and service facilities), Najima Station (high-density residence), and Susenji Station (low-density residence). There is more commercial land in this area than in other areas and there is little green land in this district (upper part of map on left); the ratio of public management and service facilities in this group is higher and the residential areas are larger than in some other areas (upper part of map on right); and the proportion of residential areas in this group is much higher than that in other groups, but the ratio of park and green land in this area is not high (lower part of map on left). It can be clearly seen that few houses are in this area, as well as little commerce and few public management and service facilities, leaving a lot of green and unused land (lower part of map on right).

## 6. Discussion

### 6.1. Theoretical Contributions

Our study contributes to the body of research on Japan’s rail transit and urban planning in the following ways. 

First, we explored a new research perspective (analyzing rail transit station zones and analyzing stations by typology) to determine the characteristics of land use and the transitions of the rail transit network. Typology analysis of stations is more conducive to the study of rail transit succession and transition.

Second, our study enriches the current research literature on transportation and urban planning, providing a reference for global research on rail transit and the urban environment [63].

Third, we analyzed urban spatial succession around rail transit stations according to influence factors and cluster analysis, which are feasible methods for studying rail transit networks [64]. The results show that the methods suggested by authors not only improve the typological method on spatial analysis but also expand outcomes on station typification, changes on the transition modes and characteristics of rail transit zones. 

Furthermore, the current study spans the 30-year history of rail transit development and transportation in Japan, from the 1980s to the 2010s. This period had a far-reaching impact on urban construction and influenced rapid urban development after the war. It was a golden period of urban development, transition, and consolidation, and studying it can provide a reference for rail transit and urban planning in other countries.

### 6.2. Practical Implications

In this study we took land and building use around rail transit stations in Fukuoka as the research object, used GIS to extract POSMAP data, and compared changes according to the land use distribution. The analysis in this study comprised two steps: a radius of 0–400 m and a radius of 400–800 m. Through cluster analysis, stations were clustered into representative groups according to different characteristics. We also found out the conditions of annual periodic changes, clarified the space extension, and analyzed the typological trends and characteristics of urban spatial succession, which can provide a reference for the redevelopment of cities and sustainable development. We found that the rail transit network in Japan shows the characteristics of high accessibility and aggregation, in which a few stations are important multi-line transfer hubs, and the accessibility of the network relies on the center of rail transit network as the core decreases. In addition, commerce-type stations are highly dependent on accessibility due to the commercial potential brought by large passenger flow, commercial sub-centers are constantly forming, and peripheral commercial stations are conveniently connected with other stations in the city.

The development modes of the Japanese rail transit network and urban spatial succession have important enlightenment significance for the development of cities in other countries. It is necessary to consider both the top-level design and land use planning of rail transit networks and the node characteristics of the transportation where stations are located, so as to form a coordinated coupling mechanism. Our findings have several implications, as follows:

(1) A multi-level rail transit network was established, with a complete transportation service system. Some cities in the world only construct two-level transportation networks, where the city centers are connected by subways and railways, or cities are connected by high-speed railways, so that the starting and ending points of short-distance and long-distance intercity transportation are mixed together [65], which increases the burden of railway station hubs [66] and internal transportation in cities [67]. On the contrary, the framework designed and formed for rail transit gives it high accessibility of the small world network and improves travel efficiency [68]. In addition, Japan’s rail transit adopted several transportation services: city centers rely on subways and suburbs rely on private railways, and the high-speed Shinkansen railway starts from central stations and quickly connects to other cities across the country, making the flow of intercity travel evenly dispersed throughout JR networks, which optimizes overall network flexibility and social travel cost savings. Thus, in the future, urban transit networks can be added between city subways and the national railway network, and multi-point integration can be carried out with the subways;
(2) The integration of external transportation hubs and commercial facilities resources was strengthened. In some countries, to reduce construction costs and cycles, some new high-speed railway stations in large cities are located at the edge of the city, with a weak business atmosphere and logistics supply chain [69,70], and lack a connection to subways and main functional areas of the city [71]. On the contrary, in Japan, rail transit comprises a three-pronged combination of external transportation hubs, internal accessibility centers, and business centers so that people living in other districts can easily visit them for work and shopping. Therefore, in the process of land development, high-speed railway stations can be integrated with highly accessible areas with great commercial potential, or the high-speed transportation services of existing railway stations in city centers can be reasonably increased;
(3) Line operation of suburban rail transit and land development were optimized. In their early stages, the subways in big cities in some countries often only served the city centers. With the development of suburban land and easing of the population in city centers, an insufficient supply of suburban rail transit has become a major problem [72]. In the future, the locations of suburban rail transit lines and stations could be further optimized. Express transportation services connecting stations and city centers could be added through reasonable planning of land use along the subways, two-way passenger flow in different time periods could be balanced, and commuter congestion during peak periods could be alleviated;
(4) The traffic accessibility of new districts and other urban clusters was improved. The housing problem brought about by Japan’s bubble economy to the land price. Urban construction in Japan highly praised “one family, one house”, the rise in land prices brought about serious housing shortage. At that time, this situation led to a stampede to buy property, which further stimulated the rise in land prices. For some people living in the center of cities, they could not afford high land rent and housing rent at all, and had to move to the suburbs, so some new districts were actively developed as organic units of the multi-center spatial system and high-density urban groups in metropolitan areas, helping the old central urban areas to jointly carry the urban functions of the metropolitan area [73], promoting the optimization of the overall function and structures of the metropolitan area;
(5) Green resources were protected, and green urban spaces were built. The study found that the formation of green urban spaces around rail transit station zones has been significant in promoting increased land use. As commerce and transportation have prospered, ecological environment has gradually become the main development focus of the surrounding areas, and the future trend is increasingly obvious. As with the land use for green land and parks, the government should reserve it in advance in order to put it into environment improvement. In addition, in some commerce-oriented [74], industry-oriented [75], transit-oriented [76], and other areas, green urban spaces are still necessary. Rational utilization of green resources, repeated across a dozen environmental issues and across our diverse planet, is what will ultimately determine whether the human race is living beyond its ecological means as it pursues urban growth;
(6) The standards and quality of construction were attended to, and the promotion of rail transit and urban renewal projects was firmly carried out. Urban planning and development of rail transit stations was reasonably promoted based on changes in urban traffic conditions and the evolution of urban spatial structures. Based on this, in urban planning, high-density development and utilization of land were improved along the rail lines [77]. The mode of land use was updated, and the spatial structure was adjusted along the subways guided by the layout and planning of urban space [78]. Attention was paid to the spatial redistribution of urban population and the intersection of subway transportation and transfer hubs between subway lines to develop sub-centers of cities.

### 6.3. Limitations and Future Directions

This study is the beginning of a series of related studies. In order to make this study fully operable, further investigation is needed, which includes integrating more influence factors and calculating cumulative effects, in order to predict the influence degree and development trend of land use around stations and provide a more comprehensive way to approach sustainable development and apply rail transit around the world.

## 7. Conclusions

The development of cities is accompanied by the formation and successive transition of city centers. Cities have experienced an evolution from single-center to multi-center spaces. Due to expansion and connection at the regional level, centers of different scales have been formed, and urban systems have continuously improved and become more complex [79], with differences among the centers of different systems. With the development of urban rail transit, on the one hand, as the main artery, the transportation network connects functional areas of the city (workplaces, business districts, large residential districts), and on the other hand, it reshapes the original urban structure (external transportation hubs, important transfer nodes, etc.). Whether the center of a city’s transportation network is a business center or not and what the coupling relationship is between them is a topic worth studying. As the commercial and traffic centers of a city overlap and are differentiated, this will often lead to problems in urban planning and design. 

Hence, in terms of land use, demographic characteristics, socioeconomic status, transportation, and urban environment, this study analyzed the objective rules and interactive relationship between rail transit and urban development in Japan, then explored urban spatial succession around rail transit zones, aiming to guide the development of urban rail transit in the future, in order to realize coordination between urban rail transit and land use and hope to provide reference for relative studies.

From the regional perspective, each country (region) is at a different stage of urban development and has different political, cultural, economic, and other background factors. In the past, many years ago, in a country that had scarce land resources and developed regional three-dimensional transportation and underground spaces, the three-dimensional transportation node network was planned and constructed in Tokyo and was very forward-looking. Today, many years later, urban transportation and planning must be comprehensively considered in combination with the national conditions and three-dimensional transportation nodes of the geographical environment of different cities and regions in the stage of rapid urbanization. In the future, we can also look toward urban spatial succession and transitioning of the urban environment and explore multidisciplinary and multi-angle methods of sustainable development.

## Figures and Tables

**Table 1:** World literature on rail transit development, urban growth, and succession.

Reference	Case Study	Methodology	Data Source	Research Content
Gomez-ibanez (1985) [44]	San Diego (USA), Calgary and Edmonton (Canada)	Transit agency data/household travel survey	Descriptive statistics	In all three cities, the LRT costs more than the conventional bus service it replaced. Public transit ridership increased modestly in two of the three cities, but the costs per added rider were high.
Cervero (1995) [45]	Stockholm, Sweden	Historic overview/descriptive statistics	Official data (Census)	Stockholm’s sustainability lies in the strong rail orientation of its new towns, rather than in any kind of balanced growth or self-containment.
Cervero and Radisch (1996) [46]	San Francisco, USA	Comparative analysis/discrete choice modeling (binomial logit)	Field/mail survey	Neighborhood design practices exert their greatest influence on local shopping trips and other non-work purposes.
Kieschnick (2003) [47]	Texas, USA	Descriptive statistics/discrete choice modeling	Regression models	Regardless of choice, we strongly recommend that future research recognize that the data are likely generated by a distribution for which the mean is a nonlinear function of the regressors, and the variance is a function of the mean.
Alpkokin (2008) [48]	Istanbul, Turkey	Comparative analysis/theoretical modeling	Available data/rational basis	As the cities get larger, an urban form that diverges from a mono-centric city to a rather more complex spatial pattern of employment clusters would be expected.
Memon (2008) [49]	Hong Kong, China	Discrete choice modeling	Field/mail survey	The future research should be focused on design and planning parameters for reducing the effects of urban heat island and ultimately living in a better environment.
Whelan (2008) [50]	United Kingdom	Model/Case study analysis	Respondent survey	The evaluation is based on the development of an econometric demand model based on a large-scale survey of passenger preferences.
Crowley et al. (2009) [51]	Toronto, Canada	Descriptive statistics	Household travel survey	To examine how variations in walking distance to rapid transit
loo et al. (2010) [52]	New York City, USA, Hong Kong, China	Multiple linear regression (OIS)	Smart card data	Future research on TOD may pay more attention on examining how various aspects of station characteristics can be modified to increase railway patronage.
Cascetta and Cartenì (2013) [53]	Northern area of Naples, Italy	Comparative analysis/binomial model	Field/mail survey	The results of this research should be compared with those from other contexts as they have a potential impact for railways planning.
Oum et al. (2013) [54]	Japan	Comparative analysis/model	Efficiency index	The results indicate that the railroads are more socially efficient than airlines.
Sung (2015) [55]	Seoul, South Korea	Observations/multilevel regression models	Telephone survey data/Respondent survey	Our results indicated that walking activity is associated with Jacobs’ six conditions for urban vitality, including land use mix, density, block size, building age, accessibility, and border vacuums.
Van de Coevering et al. (2016) [56]	Amersfoort, Veenendaal, Zeewolde, The Netherlands	Cross lagged panel structural equation model	Field/mail survey	Results show that the residential built environment has a small but significant influence on car use and travel attitudes. In addition, the built environment influenced travel-related attitudes indicating that people tend to adjust their attitudes to their built environment. This provides some support for land use policies that aim to influence travel behavior.
Ewing et al. (2017) [57]	Denver, Los Angeles, San Francisco, Seattle, Washington, D.C., USA	Descriptive statistics—comparative analysis	Field/mail survey	Results suggest the potential for significant savings in TOD developments.
Sharma (2018) [58]	Bangalore, India	Hedonic price model	Panel data hedonic price model	Emerging cities can expect metro rail to substantially improve their economies and other co-benefits as long as finance can be obtained by capturing this value.
Iseki (2018) [30]	Washington, USA	Multilevel analysis/modeling approach	Respondent survey/official data	It is found that the number of households and the number of jobs within a walk shed serve as trip generating and attracting factors, respectively, in the AM peak period, but with higher positive coefficients for jobs.
Harrison et al. (2019) [59]	Johannesburg, South Africa	Comparative analysis	Respondent survey	Using six criteria—spatial transformation, mobility, affordable accommodation, jobs, and livelihoods, social demonstrate the mixed outcomes of inclusive TOD.
Ibraeva et al. (2020) [60]	California, USA	Descriptive statistics	Smart card data/official data	Therefore, TOD performance reflects the complex and multi-faceted nature of contemporary urban agglomerations, providing a challenging field of research.
De Vos (2021) [61]	Ghent, Belgium	Structural equation modeling approach	Field/mail survey	Direct and indirect effects of the built environment on travel attitudes. The effect of travel behavior on travel attitudes.
Tennøy (2022) [62]	Norway	Comparative analysis	Field/mail survey/Norwegian cities cases	The aim of the paper is to produce knowledge that is helpful for small and medium-sized cities aiming at more sustainable mobility by planning and developing land use in directions that improve the competitiveness of sustainable modes versus the private car.

**Table 2:** Outline of railway lines and stations.

Type	Line	Section	Distance (km)	Number of Stations
JR Kyushu	Kagoshima Line *	Chikuzen Shingu–Minami Fukuoka	19.8	10
	Kashii Line *	Nishi Kozaki–Doi	16.4	8 (9)
	Chikuhi Line	Susenji–Meinohama	8.1	5
	Total		44.3	23
Nishi–Nippon Railway	Tenjin Omuta Line	Nishitetsu Fukuoka Kuko	8	7
	Kaizuka Line	Kaizuka–Mitoma	9	9
	Total		17	16
Fukuoka Subway	Line 1 (Kuko Line)	Meinohama–Fukuoka Kuko	13.1	13
	Line 2 (Hakozaki Line)	Nakasu Kawabata–Kaizuka	4.7	6 (7)
	Line 3 (Nanakuma Line)	Hashimoto–Tenjin Minami	12	16
	Total		29.8	35
Total			91.1	74

* Kashii Station is on both Kagoshima and Kashii Lines.

**Table 3:** Changes in station zone acreage and population.

Change in Station Zones	1985	1995	2000	2005	2010
Population	552,526	710,932	745,002	966,883	1,066,772
Ratio of population (%)	47.6	55.3	55.5	69	72
Area (km^2^)	89.7	104.1	108.4	136.4	156.6
Ratio of total area (%)	26.6	30.8	31.9	40.1	42
Urban density (person/km^2^)	6160	6829	6873	7089	6812
Ratio of high-density residential area (%)	67.2	70.5	72.7	90.7	93

**Table 4:** Changes in building use around station zones (%).

Station Zone	Year	Business and Hotel	Entertainment	Detached House	Condominium	Government/ Education	Transportation	Industry	Plot Ratio	PopulationDensity
		%								Person/km^2^
0~400 m	1985	15.5	6.4	30.0	23.2	17.4	2.9	3.6	52.4	5240
	1993	15.7	8.1	19.2	35.5	16.8	2.4	2.2	56.0	5600
	1998	14.7	6.6	18.3	43.1	12.9	2.0	1.5	54.1	5410
	2003	14.9	7.2	16.7	43.6	12.8	2.4	1.4	58.3	5830
	2008	14.6	7.8	13.3	45.2	10.1	2.2	1.2	59.2	5920
400~800 m	1985	10.1	3.1	33.9	25.5	11.5	9.1	5.5	37.0	3700
	1993	8.5	3.9	24.7	36.9	12.7	7.0	4.9	41.8	4180
	1998	9.6	4.1	23.6	43.2	8.7	5.9	3.5	38.6	3860
	2003	9.5	4.9	22.2	42.6	8.9	7.0	3.2	37.8	3780
	2008	9.8	4.7	20.0	48.5	7.9	7.8	2.9	26.5	2650
800+ m	1985	7.4	3.7	43.3	20.0	11.9	6.9	4.3	9.1	910
	1993	6.3	4.1	38.8	26.4	12.9	4.7	4.0	11.3	1130
	1998	7.7	4.1	38.1	30.5	9.1	4.9	2.9	12.2	1220
	2003	7.2	5.0	36.6	30.9	9.3	4.7	2.9	11.9	1190
	2008	7.5	5.6	34.4	32.1	9.8	4.8	2.7	11.2	1120

**Table 5:** Changes in development amount and distribution around station zones.

Station Zone	Building Usage	1994		1996		1998		2000		2002		2004	
		DA	%	DA	%	DA	%	DA	%	DA	%	DA	%
0–400 m	Detached house	455	48.0	464	45.2	279	37.9	286	40.9	245	39.8	223	38.3
	Condominium	309	32.6	350	34.1	293	39.8	269	38.5	273	44.3	276	47.4
	Store	39	4.1	46	4.5	33	4.5	28	4.0	21	3.4	19	3.3
	Restaurant	11	1.2	20	1.9	15	2.0	14	2.0	7	1.1	6	1.0
	Office	89	9.4	75	7.3	69	9.4	60	8.6	36	5.8	22	3.8
	Total	948		1026		736		699		616		582	
400–800 m	Detached house	774	57.5	765	56.1	574	54.5	599	53.4	427	46.4	382	47.4
	Condominium	407	30.2	437	32.1	348	33.0	401	35.7	353	38.4	320	39.7
	Store	19	1.4	30	2.2	29	2.8	22	2.0	28	3.0	26	3.2
	Restaurant	12	0.9	15	1.1	10	0.9	15	1.3	11	1.2	12	1.5
	Office	76	5.6	51	3.7	46	4.4	50	4.5	52	5.7	56	6.9
	Total	1346		1363		1054		1122		920		806	
800+ m	Detached house	1820	69.4	1947	70.5	1251	61.5	1244	67.2	1107	66.9	1008	66.9
	Condominium	515	19.6	485	17.6	427	21.0	386	20.8	363	21.9	343	22.8
	Store	47	1.8	62	2.2	40	2.0	43	2.3	33	2.0	30	2.0
	Restaurant	12	0.5	26	0.9	13	0.6	24	1.3	17	1.0	13	0.9
	Office	112	4.3	104	3.8	77	3.8	70	3.8	53	3.2	49	3.3
	Total	2623		2763		2033		1852		1654		1506	

DA, development amount.

**Table 6:** Annual change rate of development areas around station zones (%).

Station Zone	1994	1996	1998	2000	2002	2004	2006	2008	2010
0–400 m	27.0	28.1	30.2	29.9	34.1	33.2	32.1	35.3	34.3
400–800 m	27.4	37.9	28.3	31.7	30.3	34.2	33.2	30.6	31.6
800+ m	45.5	33.9	41.5	38.4	35.6	32.1	36.3	40.2	42.2

**Table 7:** Total variance explained.

Component	Initial Eigenvalues			Extraction Sums of Squared Loadings			Rotation Sums of Squared Loadings		
	Total	% of Variance	Cumulative %	Total	% of Variance	Cumulative %	Total	% of Variance	Cumulative %
1	3.478	26.756	26.756	3.478	26.756	26.756	3.467	26.666	26.666
2	2.018	15.526	42.282	2.018	15.526	42.282	2.008	15.444	42.110
3	1.394	10.722	53.004	1.394	10.722	53.004	1.264	9.725	51.835
4	1.274	9.799	62.803	1.274	9.799	62.803	1.225	9.420	61.255
5	1.078	8.290	71.092	1.078	8.290	71.092	1.202	9.244	70.499
6	1.008	7.752	78.844	1.008	7.752	78.844	1.085	8.344	78.844
7	0.937	7.208	86.052						
8	0.738	5.676	91.728						
9	0.622	4.783	96.511						
10	0.354	2.721	99.232						
11	0.069	0.532	99.764						
12	0.028	0.212	99.976						
13	0.003	0.024	100.000						

Extraction method: principal component analysis.

**Table 8:** Component transformation matrix.

Component	1	2	3	4	5	6
1	0.998	−0.009	−0.042	−0.023	−0.049	−0.003
2	0.002	0.994	−0.071	−0.056	−0.055	−0.003
3	0.002	0.008	0.782	−0.457	−0.405	−0.122
4	−0.013	0.006	0.085	0.633	−0.666	0.384
5	0.039	0.070	0.359	0.598	0.223	−0.676
6	0.056	0.078	0.495	0.170	0.580	0.617

Component **1**: commerce; component **2**: house; component **3**: public management and service; component **4**: park and green land; component **5**: transportation and roads; component **6**: industry and others.

**Table 9:** Station classification in 0–400 m radius after cluster analysis.

Type	Stations
Group 1: Commerce	Nishitetsu Fukuoka, Yakurin, Nishitetsu Hirao, Nishitetsu Kashii, Kashii Harazonomae, Akasaka, Tenjin, Nakasu Kawabata, Gion, Hakata, Higashi Hire
Group 2: Public management and service	Chikuzen Shingu, Kyusandaimae, Saitozaki, Takaraha, Gofukumachi, Chiyokenchiguchi, Maidashi Kyudai Byoin Mae, Hakozaki Kyusandai Mae
Group 3: High-density residence	Kashii, Meinohama, Ganosu, Doi, Ijiri, Najima, Hshii Gumae, Tonahara, Mitoma, Fujisaki, Nishijin
Group 4: Medium-density residence	Yoshitsuka, Takeshita, Sasahara, Minami Fukuoka, Shinoyamamoto, Nata, Wajiro, Ohashi, Kaizuka, Hakozaki Miyamae
Group 5: Low-density residence and industry	Chihaya, Hakozaki, Imajuko, Susenji, Zayonokuma, Fukuoka Kuko
Group 6: Park and green land	Mai Matsubara, Nishitetsu Chihaya, Tojinmachi, Ohori Koen

**Table 10:** Result of cluster analysis in 0–400 m radius (including land use, population, and passengers).

Cluster	Station	Line	Land Use						Passenger (Person/ Day)	Population (Person)
			Commerce	House	Public Management & Service	Park & Green Land	Transportation & Road	Industry & Others		
			(%)							
	**Type of Commerce**									
	Nishitetsu Fukuoka Tenjin	Railway	29.8	26.8	5.4	0.7	24.6	12.6	50,188	7176
	Yakuin	Railway	16.8	46.3	6.6	1.2	21.7	7.4	12,678	17,368
	Nishitetsu Hirao	Railway	6.2	59.7	4.1	2.1	22.0	5.9	4348	22,901
	Nishitetsu Kashii	Railway	16.3	45.0	5.2	2.2	16.4	14.9	1170	16,441
11	Nishitetsu Kashiikaenmae	Railway	31.2	12.5	31.9	1.9	13.8	8.7	967	7595
	Akasaka	Subway	27.6	31.5	13.6	0.1	20.6	6.7	9628	14,234
	Tenjin	Subway	40.1	12.1	8.2	5.2	28.2	6.2	43,591	6305
	Nakasu-kawabata	Subway	38.9	14.2	10.3	3.2	25.8	7.6	9353	6439
	Gion	Subway	32.5	9.2	10.3	0.9	29.4	17.7	4431	9395
	Hakata	Subway	39.2	6.6	7.1	4.1	24.0	19.0	39,699	8758
	Higashi-hie	Subway	28.0	18.7	1.3	1.2	25.7	25.2	5663	9407
	**Average**		27.9	25.7	9.5	2.1	22.9	12.0	16,520	11,456
	**Type of Public management and service**									
	Chikuzenshingu *	JR	0.8	45.7	19.6	4.4	16.4	13.1	8177	8338
	Kyusandai-mae	JR	4.11	30.4	42.6	1.7	16.5	4.6	4549	10,955
	Saitozaki	JR	0.7	47.4	17.6	2.2	10.8	21.4	613	2498
	Takamiya	Railway	8.1	37.2	22.2	4.1	22.1	6.3	6843	22,368
8	Gofukumachi	Subway	23.1	14.4	27.0	2.2	23.5	10.0	1986	12,661
	Chiyo-kenchoguchi	Subway	19.3	20.7	26.4	0.8	25.4	7.4	2315	15,624
	Maidashi-kyudaibyoinmae	Subway	6.6	17.5	32.6	15.7	20.3	7.4	2912	10,017
	Hakozaki-kyudaimae	Subway	8.9	47.9	13.9	5.2	20.5	3.7	2325	15,080
	**Average**		8.9	32.7	25.2	4.5	19.4	9.2	3425	12,193
	**Type of High-density residence**									
	Kashii	JR	1.3	60.0	10.1	2.8	13.1	12.8	8493	13,842
	Meinohama	JR	13.3	59.7	3.2	10.0	0.0	13.7	4259	16,329
	Gannosu	JR	2.3	60.6	0.1	0.4	15.9	20.6	244	3985
	Kashiijingu	JR	2.3	52.1	3.5	9.1	16.3	16.7	645	12,879
	Doi	JR	8.2	58.4	0.7	3.5	1.0	28.2	802	7714
13	Ijiri	Railway	7.1	52.6	2.4	1.0	16.6	20.4	7986	17,218
	Najima	Railway	1.2	53.4	5.8	5.0	13.6	21.0	629	11,714
	Kashiimiyamae	Railway	2.4	53.1	2.9	2.5	17.0	22.1	880	13,713
	Tonoharu	Railway	3.7	52.1	1.0	1.1	19.6	22.6	380	12,058
	Mitoma	Railway	4.3	52.2	3.7	3.2	17.6	19.0	1184	10,303
	Muromi	Subway	4.7	64.0	7.1	0.4	20.6	3.1	5154	16,831
	Fujisaki	Subway	5.1	60.7	6.2	1.9	19.5	6.5	7316	17,242
	Nishijin	Subway	16.6	50.8	3.8	0.8	21.7	6.3	14,910	19,607
	**Average**		5.6	56.1	3.9	3.2	14.8	16.4	4068	13,341
	**Type of Medium-density residence**									
	Yoshizuka	JR	12.9	38.9	14.2	1.7	17.2	15.1	7178	15,693
	Takeshita	JR	3.6	41.9	9.6	1.6	17.2	26.0	3696	11,406
	Sasabaru	JR	4.2	51.6	2.2	4.1	19.6	18.3	2694	14,703
	Minami-Fukuoka	JR	4.7	47.5	13.2	2.0	20.4	12.2	6113	7591
10	Shimoyamato	JR	3.2	45.7	9.2	1.5	14.8	25.7	1561	10,319
	Nata	JR	0.0	50.8	11.4	2.1	19.5	16.2	875	11,235
	Wajiro	JR	5.9	50.2	7.6	0.8	14.4	21.1	1110	9467
	Ohashi	Railway	8.4	46.4	7.3	1.0	23.4	13.5	12,596	20,859
	Kaizuka	Subway	6.4	46.3	1.1	10.7	22.3	13.2	4587	13,639
	Hakozaki-miyamae	Subway	8.3	43.5	11.5	2.7	22.3	11.7	2210	4233
	**Average**		5.8	46.3	8.7	2.8	19.1	17.3	4262	11,915
	**Type of Low-density residence and industry**									
	Chihaya	JR	4.2	31.5	12.1	2.0	13.9	36.4	5128	12,488
	Hakozaki	JR	14.8	33.0	8.3	1.7	8.9	33.3	2992	8675
6	Imajuku	JR	6.3	38.2	0.5	0.4	11.8	42.8	3119	8027
	Susenji	JR	1.4	10.2	0.9	0.1	5.9	81.6	3430	8016
	Zassyonokuma	Railway	15.9	36.5	6.5	0.6	21.6	18.9	5653	13,806
	Fukuokakuko (Airport)	Subway	3.9	38.0	6.0	9.4	15.5	27.3	14,343	2112
	**Average**		7.7	31.2	5.7	2.4	12.9	40.0	5778	8854
	**Type of Park and green land**									
	Maimatsubara	JR	3.8	52.5	1.3	6.2	18.5	17.8	664	10,660
4	Nishitetsu Chihaya	Railway	3.9	26.6	12.8	2.0	12.1	42.7	1208	12,499
	Tojinmachi	Subway	2.4	27.9	3.2	21.5	11.6	33.4	6367	14,599
	Ohorikoen	Subway	4.6	7.5	14.7	40.2	18.2	14.8	5394	17,188
	**Average**		3.7	28.6	8.0	17.4	15.1	27.1	3408	13,737

* The name of Chikuzenshingu Station has been changed into Fukkodai-mae Station in 2008.

## Data Availability

Not applicable.
